# The Treatment of Latent Tuberculosis

**Published:** 1905-04-01

**Authors:** 


### THE TREATMENT OF LATENT TUBERCULOSIS.

Dr. Arthur Latham[1] insists upon the doctrine that hygienic treatment, although not a specific in pulmonary tuberculosis, affords a better prospect of cure than any other method. To obtain its full value, however, the patient must be brought under its influence when the disease is yet in an early stage. Hence arises the question as to the means by which the recognition of commencing pulmonary phthisis can be facilitated. When there are definite physical signs or when the sputum contains tubercle bacilli there is no doubt about the diagnosis, but equally there is no doubt that the disease has become firmly established and that its extent is considerably wider than the physical signs taken by themselves suggest. Hence to delay hygienic or sanatorium treatment until one or other of these conditions is satisfied is to pursue a policy which avoids advantage and invites risk. Therefore it is obvious that if the full benefit of the sanatorium treatment is to be obtained the patient in many instances must be placed under its influence while the diagnosis of pulmonary tubercle is more or less uncertain. To this it may be objected that such a course is not justified, and further that it involves a risk that a non-tubercular patient may become infected by association with undoubted victims of pulmonary tubercle. The reply to these positions is, first, that whilst the diagnosis may not be absolute, there yet may be good reason for suspicion and that in such circumstances the pathway of safety is prompt and thorough treatment, and, secondly, that a properly conducted sanatorium offers less risk of infection than does an ordinary hotel. There seems every reason to believe that between the date of infection in any given case and the appearance of physical signs a very considerable time—perhaps

18 months or two years—may elapse, and this consideration emphasises the need for treatment even when the diagnosis can hardly claim to be other than a more or less definite suspicion. Among circumstances justifying such suspicion a prominent position must be given to hæmoptysis. Spitting of blood for which no explanation can be discovered ought to be regarded as due, in all probability, to pulmonary tubercle, and without waiting for confirmatory evidence the patient should be placed under the discipline and training of a sanatorium. An identical position should be maintained in reference to pleurisy occurring in children or adolescents and not capable of reference to a pneumonic, a streptococcic or a rheumatic causation. Here a further fact strengthens this advice, namely, that cases having a pleuritic origin are usually favourable ones, and if treated usually give most gratifying results. A third group to which similar advice should be tended includes patients who, with a history of digestive disturbance, complain of slight wasting, cough, and lassitude. And in favour of this plan it may in addition be urged that treatment when commenced early will need a relatively brief continuance and is more likely to secure a permanently beneficial result. In all such instances the continuous observation possible at a sanatorium is much more likely to lead to a definite diagnosis than is a mere occasional examination. Hence if such observation proves adverse to the theory of tubercle no harm has been done, whilst if the opposite conclusion is indicated the treatment has commenced at a time when the chances of success are at the highest level. A further point of practical importance depends on the relationship of disease of the upper air passages to the development of tuberculosis. The probabilities are that tubercle bacilli cannot pass through the nasal spaces unless these are the site of disease, and that the usual channel of infection is either through the nose in the circumstances just indicated or through the mouth and thence by the lymphatics to the lungs. Hence it is advisable when disease of the upper air passages is known to exist to keep a specially sharp look out for evidences of tuberculosis, and if they are discovered to place the patient at once under hygienic treatment. Many cases of tuberculosis owe their origin to the condition of the upper air passages, and the recognition of this fact would mean a more ready appreciation in such instances of pulmonary tubercle while in the latent stage.

[1] La Revue Internationale de la Tuberculose.

